# Facile Synthesis of N-Doped Graphene-Like Carbon Nanoflakes as Efficient and Stable Electrocatalysts for the Oxygen Reduction Reaction

**DOI:** 10.1007/s40820-017-0181-1

**Published:** 2017-12-27

**Authors:** Daguo Gu, Yao Zhou, Ruguang Ma, Fangfang Wang, Qian Liu, Jiacheng Wang

**Affiliations:** 1 School of Materials Engineering, Yancheng Institute of Technology, Yancheng, 224051 Jiangsu Province People’s Republic of China; 2 State Key Laboratory of High Performance Ceramics and Superfine Microstructure, Shanghai Institute of Ceramics, Chinese Academy of Sciences, 1295 Dingxi Road, Shanghai, 200050 People’s Republic of China; 3 Shanghai Institute of Materials Genome, 99 Shangda Road, Shanghai, 200444 People’s Republic of China

**Keywords:** Nitrogen doping, Graphene-like, Carbon nanoflakes, Electrocatalyst, Oxygen reduction reaction

## Abstract

A series of N-doped carbon materials (NCs) were synthesized by using biomass citric acid and dicyandiamide as renewable raw materials via a facile one-step pyrolysis method. The characterization of microstructural features shows that the NCs samples are composed of few-layered graphene-like nanoflakes with controlled in situ N doping, which is attributed to the confined pyrolysis of citric acid within the interlayers of the dicyandiamide-derived g-C_3_N_4_ with high nitrogen contents. Evidently, the pore volumes of the NCs increased with the increasing content of dicyandiamide in the precursor. Among these samples, the NCs nanoflakes prepared with the citric acid/dicyandiamide mass ratio of 1:6, NC-6, show the highest N content of ~6.2 at%, in which pyridinic and graphitic N groups are predominant. Compared to the commercial Pt/C catalyst, the as-prepared NC-6 exhibits a small negative shift of ~66 mV at the half-wave potential, demonstrating excellent electrocatalytic activity in the oxygen reduction reaction. Moreover, NC-6 also shows better long-term stability and resistance to methanol crossover compared to Pt/C. The efficient and stable performance are attributed to the graphene-like microstructure and high content of pyridinic and graphitic doped nitrogen in the sample, which creates more active sites as well as facilitating charge transfer due to the close four-electron reaction pathway. The superior electrocatalytic activity coupled with the facile synthetic method presents a new pathway to cost-effective electrocatalysts for practical fuel cells or metal–air batteries. 
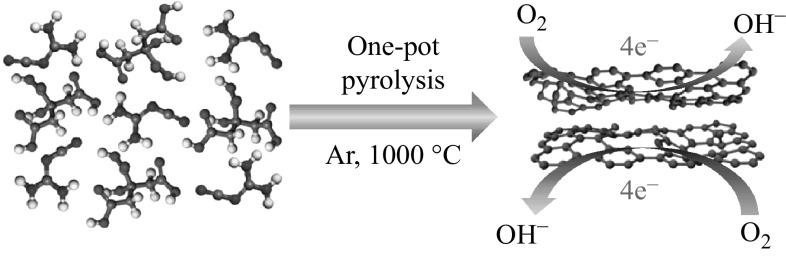

## Highlights

- N-doped carbon nanoflakes (NCs) were synthesized via facile one-pot pyrolysis of citric acid and dicyanamide.
- The as-synthesized NCs have a stacked, few-layered graphene-like structure enriched with pyridinic and graphitic N groups.
- The optimized NCs show better long-term stability and highly active in electrocatalytic oxygen reduction with a close four-electron reaction pathway.

## Introduction

Oxygen electrochemistry plays an important role in the development of advanced energy storage systems, such as metal–air batteries and fuel cells [1, 2]. Owing to the high overpotential in the oxygen reduction reaction (ORR), it is necessary to employ an effective catalyst at the cathode [3]. To date, the most commonly used catalysts for this purpose are based on noble metals, such as commercial 20 wt% Pt/C, which is quite expensive [4, 5]. In order to successfully replace the noble metals, the substitutes must satisfy the following requirements: (1) efficient catalytic activity comparable to that of commercial Pt/C, (2) excellent long-term stability to resist the methanol/CO poisoning in the electrolyte, (3) low cost, i.e., high abundance in the earth’s crust, and (4) facile synthesis. Consequently, many efforts have been devoted to developing eligible candidates, including Pt-alloys with inexpensive metals such as Fe and Ni. [6, 7], transition metal oxides [8, 9], carbides and nitrides [10], carbon-based materials [11–14], and so on [15, 16].

Among the alternative electrocatalysts mentioned above, carbon-based materials such as heteroatom-doped graphene [17–19], graphitic carbon nitride [20], and carbon nanotubes [21, 22], possess unique advantages in terms of abundance, stability, and scalability. In particular, N-doped carbon materials (NCs) exhibit comparable or superior electrocatalytic activity to Pt-based catalysts and also demonstrate high potential in the practical application of ORR-related devices [23–26]. Upon N doping, the spin density and charge distribution of carbon atoms will be influenced by the neighboring N atoms, which effectively weaken the O–O bonding and facilitate electron transfer on the carbon materials. On the other hand, it has been reported that increasing the specific surface area (SSA) by introducing porosity can expose as many active sites as required for a particular mass loading, which is another effective strategy to improve the electrocatalytic activity [27].

To obtain the desired NCs, numerous synthetic methods have been developed. Typical N-doped ordered mesoporous carbon materials were synthesized by Wang and co-workers under flowing NH_3_ at high temperatures (e.g., 950–1050 °C), which exhibited a significant ORR activity in acidic media [28]. In the presence of ammonia, N-doped graphene has also been harvested by chemical vapor deposition of methane [29]. Alternatively, Jeong et al. prepared N-doped graphene by a modified Hummer’s method followed by a nitrogen plasma process [30]. The solvothermal method is a facile and mild synthetic approach to produce N-graphene or NCs by utilizing CCl_4_, metallic K, and N-containing precursors as starting materials at an appropriate temperature [31, 32]. Commonly, NCs can be obtained by a two-step method including first synthesizing carbon materials and then heat-treating the as-prepared carbon materials in N-containing atmospheres at high temperatures.

**Scheme 1:**
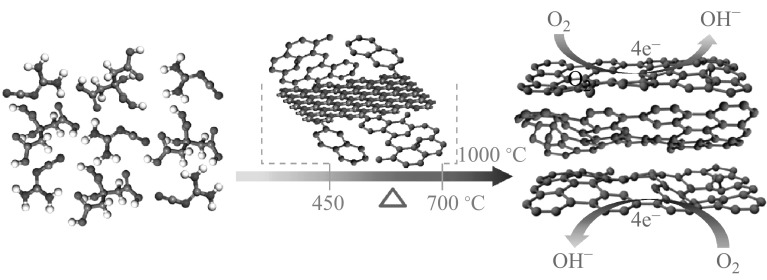
Synthesis of nitrogen-doped graphene-like carbon nanoflakes by pyrolyzing biomass citric acid and dicyandiamide at 1000 °C

Despite the great progress in their synthesis, a facile and scalable method is still highly appealing for the fabrication of NCs in one step, including the in situ N doping. Herein, we developed a facile one-step synthetic approach to obtain N-doped graphene-like carbon nanoflakes by simultaneously pyrolyzing biomass citric acid and dicyandiamide as renewable materials at 1000 °C for 1 h, as illustrated in Scheme 1. During pyrolysis, dicyandiamide formed two-dimensional (2D) graphitic carbon nitride (g-C_3_N_4_) nanosheets at 450–700 °C [33], which were then used as an in situ template for the confined growth of 2D carbon nanoflakes from citric acid. At temperatures higher than 700 °C, g-C_3_N_4_ started to decompose and N doping occurred simultaneously to form the NCs with a flake-like morphology [33, 34]. The derived g-C_3_N_4_ played two important roles in the formation of NCs, it acted as a nitrogen source and as the in situ template [35]. The as-prepared NCs exhibited not only comparable ORR activity to commercial 20 wt% Pt/C, but also superior long-term durability and methanol crossover resistance.

## Experimental

### Chemicals

Citric acid, potassium hydroxide, and dicyandiamide were obtained from Sinopharm Chemical Reagent Co., Ltd. Commercial Pt/C catalyst (20 wt%) was procured from Johnson Matthey (UK). Nafion^®^ solution (5%) was purchased from Sigma-Aldrich. All chemicals were used as received without further purification.

### One-Pot Synthesis of N-Doped Graphene-Like Carbon Nanoflakes

In a typical synthetic procedure, citric acid (1 g) was dissolved in deionized (DI) water under vigorous stirring to form a homogeneous solution **A** at room temperature. Next, dicyandiamide was mixed with DI water in the water/dicyandiamide mass ratio of 20 to obtain a homogeneous solution **B** by heating at 100 °C. Solution **A** was then slowly added into **B** dropwise with stirring. The final mixed solution was heated at 110 °C under vigorous magnetic stirring until the water completely evaporated to yield a white solid. The product was carbonized at 1000 °C for 1 h with a ramp rate of 3 °C min^−1^ in a quartz tube furnace under argon. The resulting black solid material was ground into a fine powder for further analyses. Three samples were synthesized by changing the amount (1, 3, and 6 g) of dicyandiamide in the precursor. The final samples were designated as NC-X, where X is the mass ratio of dicyandiamide to citric acid. For comparison, pure N-free porous carbon (PC) was also synthesized by direct calcination of citric acid at 1000 °C under Ar atmosphere.

### Structural Characterization

Scanning electron microscopy (SEM) and transmission electron microscopy (TEM) images were recorded on a JEOL JSM-6700F high-resolution SEM and JEOL 2010F TEM, respectively. X-ray photoelectron spectroscopic (XPS) measurements were taken on a VG Microtech ESCA 2000 using a monochromatic Al X-ray source. Raman microspectroscopy was performed using a DXR Raman microscope (Thermal Scientific Co., USA) with 532 nm excitation wavelength. The applied power of the laser was 7 mW, and the illuminated circular area was 2.1 μm in diameter. The N_2_ adsorption–desorption measurements were taken using the Quadrasorb SI surface area and pore size analyzer (Quantachrome Instruments) at 77 K. The SSAs and pore sizes were calculated using the Brunauer–Emmett–Teller (BET) and Barrett–Joyner–Halenda (BJH) methods, respectively. The SSAs were calculated by the multipoint BET method in the relative pressure range of *P*/*P*_0_ = 0.05–0.20.

### Electrochemical Experiments

First, 5 mg of the NC-X sample was ultrasonically dispersed in 1 mL of the solvent (alcohol/DI = 1:1 in volume) with 25 µL of the Nafion^®^ solution (Aldrich, 5%) to obtain a homogeneous catalyst ink. Then, 20 µL of the NC ink solution was transferred onto a glassy carbon (GC) disk electrode (2.5 mm in diameter, Pine Research Instrumentation) using a microsyringe. The electrode was dried at 40 °C under vacuum for 30 min. The electrodes for rotating ring-disk electrode (RRDE) voltammogram measurements were prepared on a 3-mm diameter GC disk electrode (Pine Research Instrumentation). Following the same procedure, 20 wt% Pt/C ink was prepared and introduced onto the GC electrode for comparison. Electrochemical measurements were taken on a computer-controlled potentiostat (CHI 760C, CH Instrument) with a three-electrode cell equipped with gas flow systems. The GC electrodes with NC-X or Pt/C catalysts were used as the working electrodes, while saturated calomel electrode (SCE) was employed as the reference electrode and a platinum wire acted as the counter electrode. The measured potentials (vs. SCE) were converted to the reversible hydrogen electrode (RHE) scale according to the Nernst equation: $E_{\text{RHE}} = E_{\text{SCE}} + \, 0.0591 \, \times {\text{ pH }} + E^{*}_{\text{SCE}}$, where *E*_SCE_ is the experimentally measured potential (vs. SCE) and $E^{*}_{\text{SCE}}$ is equal to 0.2415 V at room temperature.

Cyclic voltammetry (CV) curves were measured in an aqueous solution of 0.1 M KOH with saturated N_2_ or O_2_ gas at the scan rate of 50 mV s^−1^. Linear scanning voltammetry (LSV) curves for the rotating disk electrode (RDE) measurements were recorded at different rotating rates varying from 400 to 2025 rpm with a scan rate of 10 mV s^−1^. The number of electrons transferred (*n*) was calculated from the LSV curves according to the Koutecky–Levich equation,

1 $$J^{ - 1} = J_{\text{k}}^{ - 1} + \left( {B\omega^{1/2} } \right)^{ - 1}$$

2 $$B = 0.2nFD_{{{\text{O}}_{2} }}^{2/3} \upsilon^{ - 1/6} C_{{{\text{O}}_{2} }}$$

where *J* and *J*_k_ refer to the measured current density and kinetic-limiting current density, respectively, *n* is the electron transfer number, *F* is the Faraday constant (96,485 F mol^−1^), *υ* is the viscosity of the electrolyte (0.01 cm^2^ s^−1^), $C_{{{\text{O}}_{2} }}$ stands for the concentration of O_2_ (1.2 × 10^−6^ mol cm^−3^ in 0.1 M O_2_-saturated KOH solution), and $D_{{{\text{O}}_{2} }}$ is the diffusion coefficient (1.9 × 10^−5^ cm^2^ s^−1^ in 0.1 M O_2_-saturated KOH solution) [36]. A coefficient of 0.2 was adopted when the rotating speed was expressed in rpm. In the case of RRDE measurements, the ring current (*I*_R_) and disk current (*I*_D_) were determined by using a Pt ring-disk electrode in O_2_-saturated 0.1 M KOH solution at a rotating speed of 1600 rpm with a sweep rate of 10 mV s^−1^. The Pt ring electrode was polarized at 0.2 V (vs. SCE). The peroxide (H_2_O_2_) yield and the electron transfer number (*n*) were determined by the following equations:

3 $$\% \left( {{\text{H}}_{2} {\text{O}}_{2} } \right) = 200 \times \frac{{I_{\text{R}} /N}}{{I_{\text{D}} + I_{\text{R}} /{\text{N}}}}$$

4 $$n = 4 \times \frac{{I_{\text{D}} }}{{I_{\text{D}} + I_{R} /N}}$$

where *N*, the current collection efficiency of the Pt ring electrode, is equal to 0.37. The long-term stability and methanol crossover tests were based on current–time (*i*–*t*) chronoamperometry measurements at a bias potential of 0.4 V (vs. RHE) and 1600 rpm.

## Results and Discussion

**Fig. 1:**
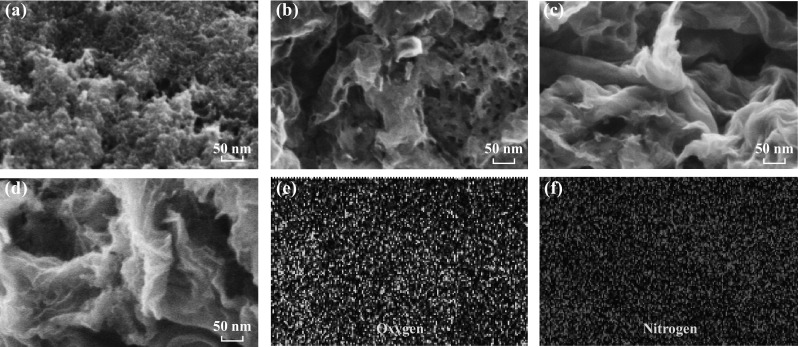
SEM images of **a** PC, **b** NC-1, **c** NC-3, **d** NC-6, and the corresponding elemental **e** O and **f** N mapping images of NC-6

**Fig. 2:**
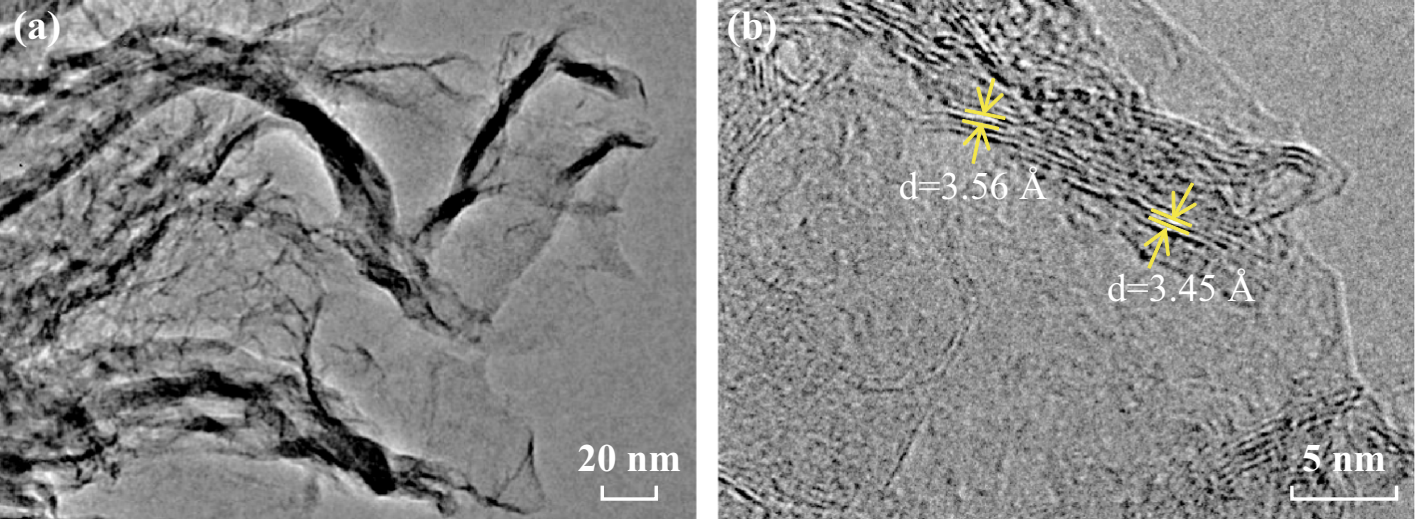
**a** TEM and **b** high-resolution TEM images of NC-6

SEM images revealed the morphological evolution of the carbon materials with the increasing ratio of dicyandiamide to citric acid, as shown in Fig. 1a–d. Without the addition of dicyandiamide in the process, the as-prepared PC had a granule-like surface, where the small particles had aggregated because of heat treatment at a high temperature (Fig. 1a). When the dicyandiamide to citric acid ratio was increased to 1:1 (Fig. 1b), the resultant NC-1 sample exhibited the flake morphology with obvious pores that were most likely a result of the gas generated from dicyandiamide decomposition. These observations indicated that the 2D carbon nanosheets were successfully prepared by the simple one-pot pyrolysis process. With further increase in the amount of dicyandiamide (3:1), the prepared NC-3 graphene-like sheets became completely crumpled [37, 38]. As shown in Fig. 1d, NC-6 had a similar morphology as that of NC-3, suggesting that the addition of more dicyandiamide (dicyandiamide/citric acid = 6:1) did not lead to any remarkable morphological change. The EDS mappings showed that the N element existed uniformly throughout the surfaces (Fig. 1e–f), which implied the successful doping of N atoms within the carbon framework by this simple strategy. The uniform existence of the O element in these carbon-based samples is consistent with the previous reports [39, 40]. The TEM image in Fig. 2 shows the wrinkled graphene-like nanosheets, which is consistent with the observations from SEM. High-resolution TEM images revealed the ordered graphitic layers, which further confirmed the graphene-like nature of NC-6. The observed lattice spacing of ~ 3.45–3.56 Å was evidently larger than the interlayer distance of graphite [39], indicating the existence of numerous defects and rearrangements.

**Fig. 3:**
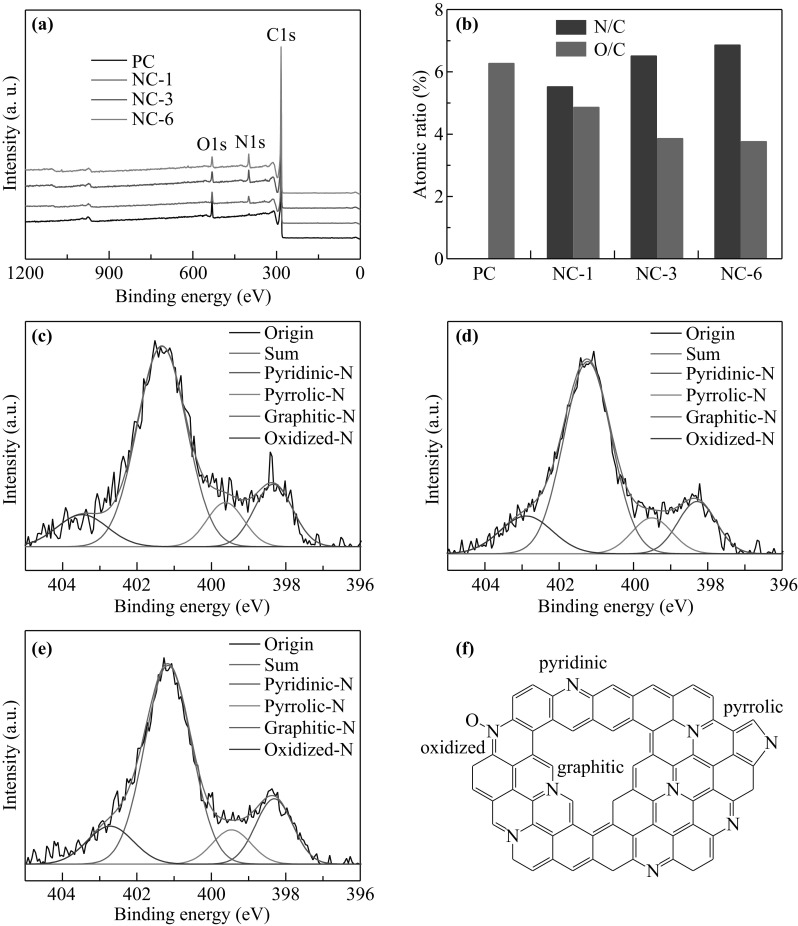
**a** XPS survey spectra of PC, NC-1, NC-3, and NC-6. **b** Atomic ratio of O/C and N/C in PC, NC-1, NC-3, and NC-6. High-resolution XPS spectra of N 1 s for **c** NC-1, **d** NC-3, and **e** NC-6. **f** Schematic illustration of N-containing species incorporated in the graphene plane of the NC-X samples

XPS is a sensitive tool to detect the elemental compositions and bonding configurations of materials. As shown in Fig. 3a, in addition to the sharp C peak, strong N and O peaks could also be observed in the spectra of NC-X samples, particularly when compared to the XPS spectrum of pure PC, indicating the successful in situ doping of N atoms [40, 41]. The O peaks could arise from the oxidized N species and the carboxyl groups at the edges, or even from the defects in the graphene-like NC-X samples. The quantitative atomic ratios of C, N, and O, the relative percentages of O/C and N/C, and the different N-containing species based on the XPS results are summarized in Table 1. Figure 3b depicts the changes in the O/C and N/C ratios by using a histogram. It clearly shows that with the increasing ratio of dicyandiamide to citric acid, the O content decreases gradually from 6.27 at% of PC through 4.86 at% of NC-1 to 3.86 at% of NC-3 and 3.76 at% of NC-6, and finally reaches a stable level when the ratio is equal to or larger than 3:1. This could be caused by the reaction between the O and N species from dicyandiamide to form gaseous NO_*x*_, which agrees well with the observed pores in the NC-X samples. On the other hand, the N/C ratio shows a direct proportion relation, increasing from 5.52 through 6.51–6.86 at%, with the increasing amount of dicyandiamide.

**Table 1:** C, O, and N content, the ratios of N/C and O/C, and relative nitrogen species analyzed by N 1 s XPS spectra of pure PC and NC-X samples

Samples	C (at%)	N (at%)	O (at%)	N/C (%)	O/C (%)	Pyridinic N (at%)	Graphitic N (at%)	Pyrrolic N (at%)	N-oxides (at%)
PC	94.1	0	5.9	0	6.27	–	–	–	–
NC-1	90.6	5.0	4.4	5.52	4.86	15.47	63.53	10.51	10.49
NC-3	90.6	5.9	3.5	6.51	3.86	15.28	62.85	9.84	12.03
NC-6	90.4	6.2	3.4	6.86	3.76	16.32	62.91	8.92	11.85

The high-resolution N 1 s spectra of NC-X (Fig. 3c–e) can be deconvoluted into four components: pyridinic N (~ 398.3 eV), pyrrolic N (~ 399.5 eV), graphitic N (~ 401.1 eV), and oxidized N (~ 402.3 eV) [40, 42–44]. The corresponding atomic structure is illustrated in Fig. 3f. The relative contents of the various N-functional groups for NC-X are given in Table 1. It can be seen that the graphitic N species are the majority (> 75%), followed by pyridinic N, oxidized N, and pyrrolic N groups in a decreasing order. This result indicates that most of the N atoms bond with C atoms in the basal plane of the graphene-like NCs. Heat treatment at high temperature (> 600 °C) leads to the transformation of the pyrrolic N to pyridinic N and graphitic N groups [12, 45, 46]. Such covalent bonding configuration exerts significant influence on the electronic structures of the adjacent carbon atoms and is responsible for the variation in catalytic activity. The O–O bond is weakened because of the bonding of O_2_ with nitrogen and/or the adjacent carbon atom, which facilitates the reduction of O_2_ [45].

**Fig. 4:**
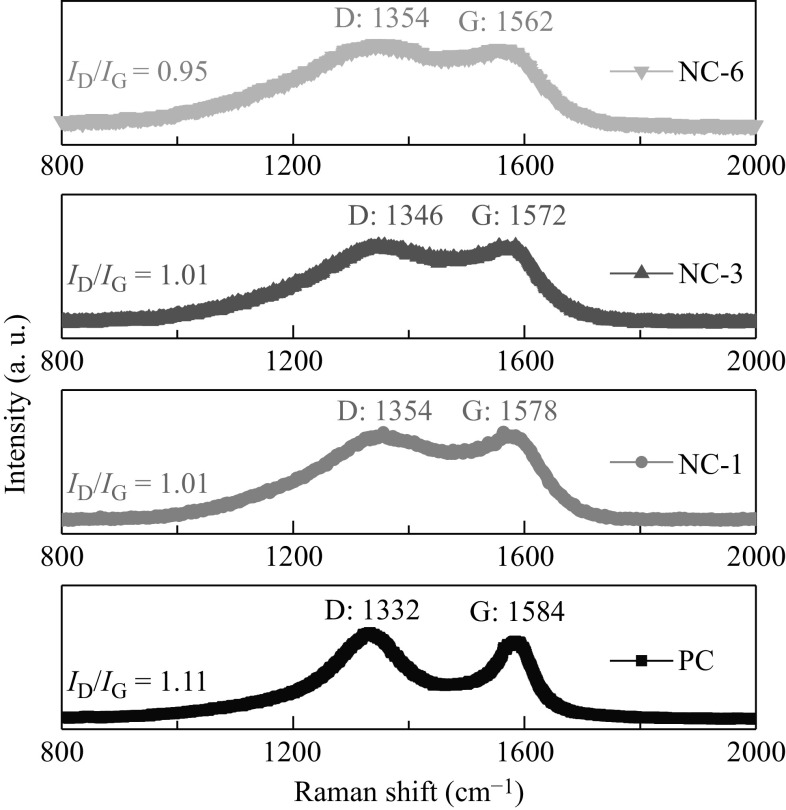
Raman spectra of the PC and NCs samples

Further structural information of the as-prepared PC and NC-X samples was obtained from their Raman spectra (Fig. 4). A typical D band resulting from the disordered carbon atoms and a G band from the *sp* ^2^-hybridized graphitic carbon atoms could be observed roughly in the range of 1332–1354 and 1584–1562 cm^−1^, respectively. The intensity ratio of the bands (*I*_D_/*I*_G_) indicated the graphitization degree of the as-prepared carbon materials. The calculated *I*_D_/*I*_G_ values for PC, NC-1, NC-3, and NC-6 were 1.1, 1.01, 1.01, and 0.95, respectively. The *I*_D_/*I*_G_ value of PC was evidently larger than that of the NC-X samples, indicating a lower degree of graphitization of PC. Furthermore, the downshift of the G peak in the samples with the increasing amount of dicyandiamide is consistent with the previous report, signifying the successful doping of N into the carbon framework of the NC-X samples [47]. The high graphitization degree of the NC-X samples is advantageous for enhancing the electrical conductivity, and thus improving the ORR catalytic activity.

**Fig. 5:**
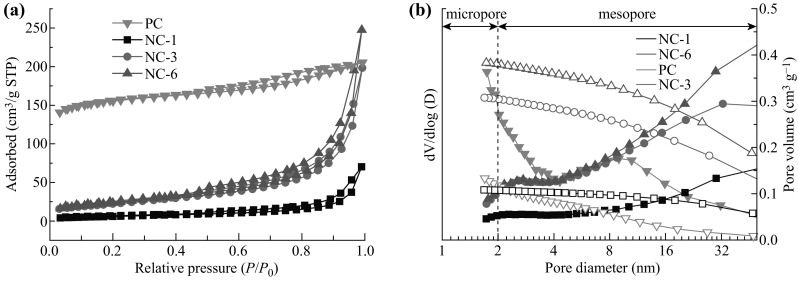
**a** N_2_ adsorption–desorption isotherms and **b** the corresponding pore size distribution curves of PC and NC-X samples calculated from the adsorption branches. Solid and empty symbols correspond to the left and right axes, respectively

In order to gain more structural information, the SSAs and porosities of the samples were investigated by the N_2_ adsorption measurements performed at 77 K [48–50], as shown in Fig. 5. All NC-X samples showed similar type-IV N_2_ adsorption/desorption isotherms with a H4 hysteresis loop, suggesting that the mesopores were a dominant feature in these materials. In contrast, PC showed a type-I isotherm (Fig. 5a), which is a typical of microporous materials [13, 51–53]. The BET surface areas (*S*_BET_) for NC-1, NC-3, and NC-6 were 22, 80, and 86 m^2^ g^−1^, respectively, which was much lower than the *S*_BET_ of PC (529 m^2^ g^−1^), as shown in Table 2. The pore size distributions (PSDs) plotted in a differential (left axis) and a cumulative pore volume (right axis) for PC and NC-X samples are presented in Fig. 5b. It is worth noting that PC showed a strong PSD peak below 2 nm and had a hump at ~ 9 nm, which were attributed to the micropores and mesopores, respectively. The SSA of the micropores (*S*_micro_) was estimated to account for 414 m^2^ g^−1^ in 529 m^2^ g^−1^ by using the *t*-plot method. The total volume (*V*_total_) was 0.318 cm^3^ g^−1^, as estimated at *P*/*P*_0_ = 0.993, and the micropore volume (*V*_micro_) was ~ 0.189 cm^3^ g^−1^. In contrast, the results for the NC-X samples only indicated the presence of mesopores (~ 2.7 nm and the wider mesopores were of size 10–50 nm). The total (mesopore) volumes were in the order of NC-1 (0.109 cm^3^ g^−1^) < NC-3 (0.307 cm^3^ g^−1^) < NC-6 (0.383 cm^3^ g^−1^), which was consistent with the trend of the dicyandiamide/citric acid ratio. This implied that the addition of dicyanamide could change the pore characteristics and increase the mesopore volumes.

**Table 2:** Textural properties of pure PC and NC-X samples

Samples	*S*_BET_ (m^2^ g^−1^)^a^	*V*_total_ (cm^3^ g^−1^)^b^	*S*_micro_ (m^2^ g^−1^)^c^	*V*_micro_ (cm^3^ g^−1^)^c^	*S*_meso_ (m^2^ g^−1^)^d^	*V*_meso_ (cm^3^ g^−1^)^d^
PC	529	0.318	414	0.189	115	0.129
NC-1	22	0.109	0	0	22	0.109
NC-3	80	0.307	0	0	80	0.307
NC-6	86	0.383	0	0	86	0.383

^a^The surface areas (*S*_BET_) were calculated by the multipoint BET method at the relative pressure range of *P*/*P*_0_ = 0.05–0.20

^b^The total pore volumes (*V*_total_) were estimated at *P*/*P*_0_ = 0.993

^c^Micropore surface area (*S*_micro_) and micropore volume (*V*_micro_) were calculated using the *t*-plot method

^d^Mesopore surface area (*S*_meso_) and mesopore volume (*V*_meso_) were calculated by using *S*_meso_ = *S*_BET_ − *S*_micro_ and *V*_meso_ = *V*_total_ − *V*_micro_, respectively

**Fig. 6:**
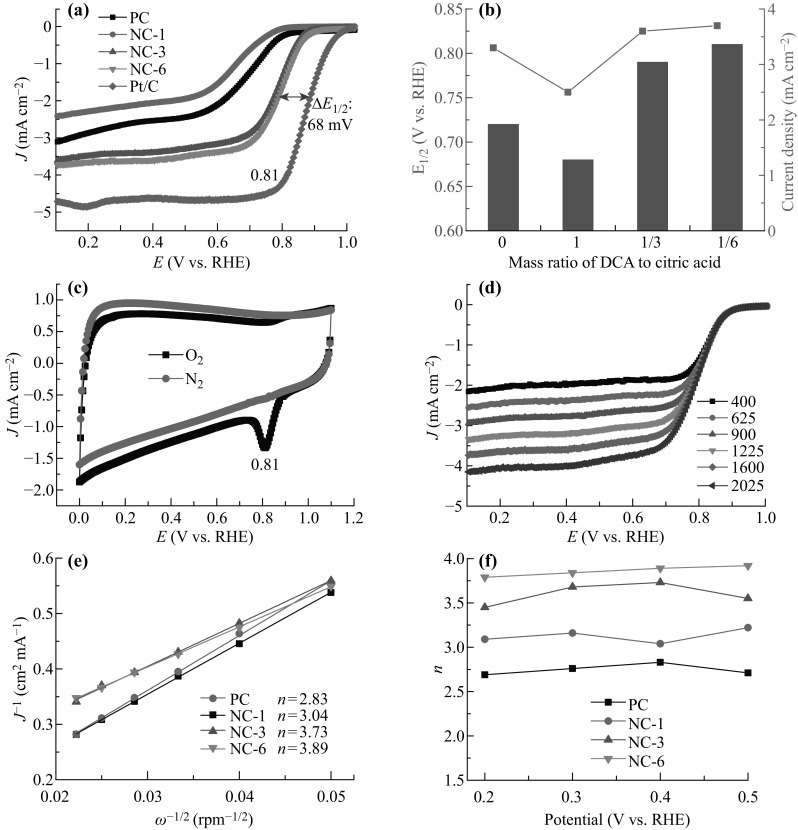
Electrochemical oxygen reduction measurements in 0.1 M KOH solution. **a** Comparison of LSV curves for PC, NC-1, NC-3, NC-6, and commercial Pt/C catalyst at a scan rate of 10 mV s^−1^ and 1600 rpm in O_2_-saturated solution. **b** Dependence of half-wave potential (*E*_half_) and current density on the mass ratio of dicyanamide (DCA) to citric acid. **c** CV curves of NC-6 in O_2_- or N_2_-saturated solution. **d** LSV curves of NC-6 and Pt/C at different rotation speeds (varying from 400 to 2025 rpm). **e** *K*–*L* plots of the samples based on the LSV curves at different rotation speeds and 0.4 V (vs. RHE). **f** Electron transfer number (*n*) calculated from the *K*–*L* plots of the various samples in the potential range of 0.2–0.5 V (vs. RHE)

The catalytic activities of the NC-X samples were first examined in a conventional three-electrode system in O_2_-saturated alkaline environment. The LSV curves of PC, NC-1, NC-3, and NC-6 at 1600 rpm clearly reveal that NC-6 exhibited the best electrocatalytic activity in terms of the half-wave potential (*E*_1/2_) and limiting current density among the as-prepared samples (Fig. 6a). The dependence of *E*_1/2_ and the current densities of the various samples on the mass ratio of dicyanamide to citric acid are shown in Fig. 6b. Compared to the commercial Pt/C catalyst, *E*_1/2_ shifted negatively to 68 mV, which is a smaller shift compared with many reported values in the literature [54–56] and similar to some latest data of N-doped materials [45, 53, 57]. Overall, considering the facile and scalable synthetic method as well as the in situ method of doping, this result is satisfactory and can be further optimized to obtain a better electrocatalytic performance. Owing to the decreasing N content in the samples, especially the pyridinic and graphitic N, the electrocatalytic activity rapidly degraded in the order of NC-1 < PC < NC-3. It is worth noting that PC exhibited better electrocatalytic activity than NC-1, which can be most likely attributed to the very high SSA of PC. Additionally, with its larger *I*_D_/*I*_G_ value and more defects compared to NC-1, PC was more prone to adsorb oxygen on the active sites and thus exhibit a better ORR activity. This observation can also be explained by the latest result reported by Qiao et al. [27], assuming that a graphene-based sample possesses a fixed doping level, the overpotential decreases with increasing SSA. Here, the SSA of PC is 24 times higher than that of NC-1 and plays a dominant role in improving the catalytic activity of PC. In other words, the best performance of NC-6 can be explained by the synergistic effect between the N-doping level and SSA. The pyridinic and graphitic N groups have been investigated as the active species for the electrocatalytic ORR [52]. Takasu et al. derived a relationship between the ORR activity and the graphitic N content [58] and concluded that the carbon atoms with Lewis basicity close to that of pyridinic N could be regarded as active sites for the ORR [56]. Other researchers found that the carbon defects could act as the ORR active sites [59]. On comparing NC-3 and NC-6, it was found that NC-6 has a smaller *I*_D_/*I*_G_ value than NC-3, although NC-3 has more defects than NC-6. However, the LSV data demonstrated that NC-6 exhibited better ORR activity than NC-3. Therefore, it was concluded that the ORR active sites are mainly derived from the N-functional groups in the NC samples.

In Fig. 6c, an obvious cathodic current with a peak centered at 0.81 V (vs. RHE) appears in the CV curve obtained in an O_2_-saturated solution, while no such a peak could be found in the N_2_-saturated solution. This indicates that the ORR takes place on the NC-6 surface. In Fig. 6d, it can be clearly seen that the current densities increase with the rotating speeds (from 400 to 2025 rpm). A sharp increase in the current density in the mixed kinetic diffusion controlled region suggests that there was an efficient diffusion of the reactants, which was facilitated by the mesoporous structure of NC-6. The subsequent current plateau pointed to the appearance of a diffusion-limiting region. The number of transferred electrons for NC-6 was calculated to be 3.89 at 0.4 V (vs. RHE) on the basis of the slope of the Koutecky–Levich (*K*–*L*) plots obtained from the LSV curves, suggesting a predominant four-electron process of ORR (Fig. 6e). This value is much larger than that of NC-3 (3.73), NC-1 (3.04), and PC (2.83), signifying that a higher amount of pyridinic and graphitic N leads to better electrocatalytic activity. Moreover, the *n* value for NC-6 in the wide potential range of 0.2–0.5 V (vs. RHE) was in the range of 3.79–3.92, also evidently larger than those for PC, NC-1, and NC-3. The four-electron process is very important for the ORR in fuel cells because the peroxides that are formed as the side products can poison the cell. The highest transfer electron number (*n*) of 3.79–3.92 of the NC-6 catalyst among the different samples was probably a result of its large surface area, high pore volume, and high N-doping level. Regardless of whether graphitic N, pyridinic N, or both N species are active sites for the ORR, there is a consensus that successful N doping is crucial for electrocatalysis. The high electronegativity of the N atoms creates a net positive charge on the adjacent carbon atoms, which changes the chemisorption mode of O_2_ from the usual end-on adsorption (Pauling model) at the nitrogen-free surface to a side-on adsorption (Yeager model) onto the N-doped electrode [60]. The N doping-induced charge transfer thus lowers the ORR potential, facilitating the ORR at the electrode.

**Fig. 7:**
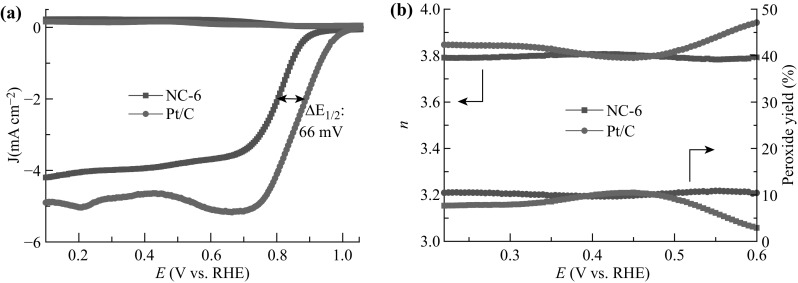
**a** Ring-disk voltammograms of NC-6 and Pt/C at a scan rate of 10 mV s^−1^ and 1600 rpm in O_2_-saturated 0.1 M KOH solution. **b** Peroxide yield and number of transferred electrons of NC-6 and Pt/C in O_2_-saturated 0.1 M KOH solution

The selectivity of the four-electron reduction of oxygen on the NC-6 catalyst was further confirmed by the RRDE technique. On the one hand, the difference of the half-wave potential (∆*E*_1/2_) compared to Pt/C is 66 mV (Fig. 7a), which is very close to the value (68 mV) measured by the RDE technique and indicates a good repetition of the NC-6 catalyst. On the other hand, the number of electrons transferred from 0.22 to 0.60 V (vs. RHE) was calculated to be ~ 3.8, as shown in Fig. 7b, which was consistent with the value (3.89) calculated from the *K*–*L* plots. This value was also comparable to the *n* value of Pt/C (3.85–3.95), illustrating a dominant 4e^−^ ORR pathway. Meanwhile, from Fig. 7b, the amount of H_2_O_2_ that reached the ring electrode was determined to be ~ 10%, which was a little higher than that of Pt/C (2.5–7.5%). Thus, NC-6 predominantly exhibited a four-electron-transfer process with a low peroxide yield for the ORR. This could be attributed to the high content of pyridinic and graphitic N, which produced more active sites and facilitated the charge transfer during the ORR process [54, 61]. Notably, the peroxides produced via a two-electron process could poison the cells by corroding the membrane and the catalyst layer [62].

**Fig. 8:**
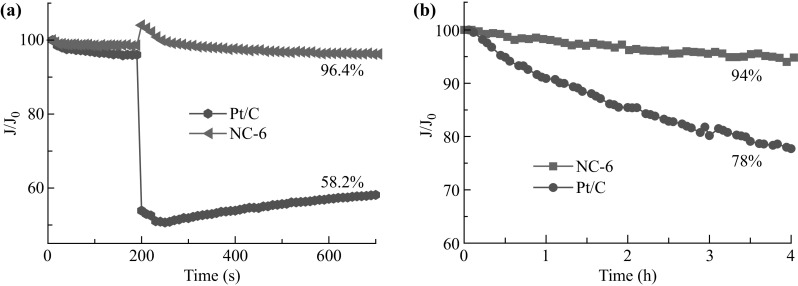
**a** Chronoamperometric responses of the NC-6 and Pt/C electrodes in 0.1 M KOH solution with 3 M methanol added after ~5 min at a rotation rate of 1600 rpm. **b** Chronoamperometric curves of NC-6 and Pt/C electrodes at 0.4 V (vs. RHE) in O_2_-saturated 0.1 M KOH solution

The resistance to the crossover effect and the long-term durability of the electrocatalysts is also of importance for their practical application in fuel cells or metal–air batteries. Therefore, the current–time (*i*–*t*) measurements were taken at 0.4 V versus RHE in O_2_-saturated 0.1 M KOH solution at 1600 rpm. When 3 M methanol was introduced into the O_2_-saturated alkaline electrolyte at 200 s, no noticeable degradation of current was observed for the NC-6 electrode, as shown in Fig. 8a, whereas a sharp drop in current occurred for the Pt/C electrode. After 500 s, NC-6 still maintained 96.4% of the kinetic current density, which was much higher than that retained using Pt/C (58.2%). As to the long-term stability, NC-6 exhibited only 6% degradation while sustaining 94% of the kinetic current density after 4 h. Thus, the stability of NC-6 was much better than that of Pt/C (78%), as shown in Fig. 8b. This could be attributed to the metal-free textures and high chemical stability of the N-relative active sites, preventing the loss of catalytic activity in the ORR. These results suggested that NC-6 with better durability and resistance to crossover effect than Pt/C, could be potentially applied in fuel cells or metal–air batteries.

## Conclusions

In summary, N-doped graphene-like few-layered carbon nanoflakes were successfully synthesized by simultaneous pyrolysis of biomass citric acid and dicyandiamide as the renewable materials at 1000 °C. During pyrolysis, the intermediate, g-C_3_N_4_, derived from the condensation of dicyandiamide, played two important roles, namely, acting as the thermally decomposable template for directing the formation of carbon nanoflakes and serving as an in situ N source upon decomposition. Compared to the commercial Pt/C catalyst, the NC flakes prepared with the dicyandiamide/citric acid mass ratio of 6 exhibited a small difference of 66–68 mV of the half-wave potential and a better long-term stability in addition to good resistance to methanol crossover, thus demonstrating an excellent electrocatalytic activity. This could be attributed to the graphitized microstructure and the high content of pyridinic and graphitic N, which presented more active sites for catalysis and also facilitated charge transfer. In terms of the ease of fabrication, low cost of biomass raw materials, and the catalytic performance, the NCs nanoflakes can be further optimized and developed as potential electrocatalysts for applications in fuel cells and metal–air batteries.

## References

[1] Park S, Shao Y, Liu J, Wang Y (2012). Oxygen electrocatalysts for water electrolyzers and reversible fuel cells: status and perspective. Energy Environ. Sci..

[2] Ge X, Sumboja A, Wuu D, An T, Li B, Goh FWT, Hor TSA, Zong Y, Liu Z (2015). Oxygen reduction in alkaline media: from mechanisms to recent advances of catalysts. ACS Catal..

[3] Dai L, Xue Y, Qu L, Choi H-J, Baek J-B (2015). Metal-free catalysts for oxygen reduction reaction. Chem. Rev..

[4] Wu G, More KL, Johnston CM, Zelenay P (2011). High-performance electrocatalysts for oxygen reduction derived from polyaniline, iron, and cobalt. Science.

[5] Cai X-L, Liu C-H, Liu J, Lu Y, Zhong Y-N (2017). Synergistic effects in CNTs-PdAu/Pt trimetallic nanoparticles with high electrocatalytic activity and stability. Nano Micro Lett..

[6] Yu W, Porosoff MD, Chen JG (2012). Review of Pt-based bimetallic catalysis: from model surfaces to supported catalysts. Chem. Rev..

[7] Song A, Yang W, Yang W, Sun G, Yin X, Gao L, Wang Y, Qin X, Shao G (2017). Facile synthesis of cobalt nanoparticles entirely encapsulated in slim nitrogen-doped carbon nanotubes as oxygen reduction catalyst. ACS Sustain. Chem. Eng..

[8] Yuan C, Wu HB, Xie Y, Lou XW (2014). Mixed transition-metal oxides: design, synthesis, and energy-related applications. Angew. Chem. Int. Ed..

[9] Guo J, Cheng Y, Xiang Z (2017). Confined-space-assisted preparation of Fe_3_O_4_-nanoparticle-modified Fe–N–C catalysts derived from a covalent organic polymer for oxygen reduction. ACS Sustain. Chem. Eng..

[10] Zhong Y, Xia X, Shi F, Zhan J, Tu J, Fan HJ (2016). Transition metal carbides and nitrides in energy storage and conversion. Adv. Sci..

[11] Shi H, Shen Y, He F, Li Y, Liu A, Liu S, Zhang Y (2014). Recent advances of doped carbon as non-precious catalysts for oxygen reduction reaction. J. Mater. Chem. A.

[12] Shang C, Li M, Wang Z, Wu S, Lu Z (2016). Electrospun nitrogen-doped carbon nanofibers encapsulating cobalt nanoparticles as efficient oxygen reduction reaction catalysts. Chemelectrochem.

[13] Chen Y, Liu Q, Wang J (2016). Highly porous nitrogen-doped carbon nanofibers as efficient metal-free catalysts toward the electrocatalytic oxygen reduction reaction. Nano Adv..

[14] Shi Q, Lei Y-P, Wang Y-D, Wang Z-M (2016). In-situ preparation and electrocatalytic oxygen reduction performance of N-doped graphene@CNF. J. Inorg. Mater..

[15] Zhou Y, Wang J (2017). Direct observation of Fe-N_4_ species as active sites for the electrocatalytic oxygen reduction. Nano Adv..

[16] Li J, Chen J, Wang H, Ren Y, Liu K, Tang Y, Shao M (2017). Fe/N co-doped carbon materials with controllable structure as highly efficient electrocatalysts for oxygen reduction reaction in Al–air batteries. Nano Adv..

[17] Wang H, Maiyalagan T, Wang X (2012). Review on recent progress in nitrogen-doped graphene: synthesis, characterization, and its potential applications. ACS Catal..

[18] Wu J, Ma L, Yadav RM, Yang Y, Zhang X, Vajtai R, Lou J, Ajayan PM (2015). Nitrogen-doped graphene with pyridinic dominance as a highly active and stable electrocatalyst for oxygen reduction. ACS Appl. Mater. Interfaces.

[19] Yu JH, Xu LL, Zhu QQ, Wang XX, Yun MJ, Dong LF (2016). Superior electrochemical performance of graphene via carboxyl functionalization and surfactant intercalation. J. Inorg. Mater..

[20] Zheng Y, Jiao Y, Chen J, Liu J, Liang J (2011). Nanoporous graphitic-C_3_N_4_@Carbon metal-free electrocatalysts for highly efficient oxygen reduction. J. Am. Chem. Soc..

[21] Tuci G, Zafferoni C, Rossin A, Milella A, Luconi L (2014). Chemically functionalized carbon nanotubes with pyridine groups as easily tunable N-decorated nanomaterials for the oxygen reduction reaction in alkaline medium. Chem. Mater..

[22] Ma TY, Dai S, Jaroniec M, Qiao SZ (2014). Graphitic carbon nitride nanosheet-carbon nanotube three-dimensional porous composites as high-performance oxygen evolution electrocatalysts. Angew. Chem. Int. Ed..

[23] White RJ, Yoshizawa N, Antonietti M, Titirici M (2011). A sustainable synthesis of nitrogen-doped carbon aerogels. Green Chem..

[24] Lin Z, Waller GH, Liu Y, Liu M, Wong C-P (2013). 3D Nitrogen-doped graphene prepared by pyrolysis of graphene oxide with polypyrrole for electrocatalysis of oxygen reduction reaction. Nano Energy.

[25] Zhou M, Wang H-L, Guo S (2016). Towards high-efficiency nanoelectrocatalysts for oxygen reduction through engineering advanced carbon nanomaterials. Chem. Soc. Rev..

[26] Xia H, Zhang J, Yang Z, Guo S, Guo S, Xu Q (2017). 2D MOF nanoflake-assembled spherical microstructures for enhanced supercapacitor and electrocatalysis performances. Nano Micro Lett..

[27] Jiao Y, Zheng Y, Davey K, Qiao SZ (2016). Activity origin and catalyst design principles for electrocatalytic hydrogen evolution on heteroatom-doped grapheme. Nat. Energy.

[28] Wang XQ, Lee JS, Zhu Q, Liu J, Wang Y, Dai S (2010). Ammonia-treated ordered mesoporous carbons as catalytic materials for oxygen reduction reaction. Chem. Mater..

[29] Qu L, Liu Y, Baek JB, Dai L (2010). Nitrogen-doped graphene as efficient metal-free electrocatalyst for oxygen reduction in fuel cells. ACS Nano.

[30] Jeong HM, Lee JW, Shin WH, Choi YJ, Shin HJ, Kang JK, Choi JW (2011). Nitrogen-doped graphene for high-performance ultracapacitors and the importance of nitrogen-doped sites at basal planes. Nano Lett..

[31] Deng D, Pan X, Yu L, Cui Y, Jiang Y (2011). Toward N-doped graphene via solvothermal synthesis. Chem. Mater..

[32] Ma R, Zhou Y, Li P, Chen Y, Wang J, Liu Q (2016). Self-assembly of nitrogen-doped graphene-wrapped carbon nanoparticles as an efficient electrocatalyst for oxygen reduction reaction. Electrochim. Acta.

[33] Wang XC, Maeda K, Thomas A, Takanabe K, Xin G, Carlsson JM, Domen K, Antonietti M (2009). A metal-free polymeric photocatalyst for hydrogen production from water under visible light. Nat. Mater..

[34] Su FZ, Mathew SC, Lipner G, Fu XZ, Antonietti M, Blechert S, Wang XC (2010). mpg-C_3_N_4_-catalyzed selective oxidation of alcohols using O_2_ and visible light. J. Am. Chem. Soc..

[35] Beckert M, Menzel M, Tölle FJ, Bruchmann B, Mülhaupt R (2015). Nitrogenated graphene and carbon nanomaterials by carbonization of polyfurfuryl alcohol in the presence of urea and dicyandiamide. Green Chem..

[36] Chen P, Wang L-K, Wang G, Gao M-R, Ge J (2014). Nitrogen-doped nanoporous carbon nanosheets derived from plant biomass: an efficient catalyst for oxygen reduction reaction. Energy Environ. Sci..

[37] Zhang M, Hou C, Halder A, Wang H, Chi Q (2017). Graphene papers: smart architecture and specific functionalization for biomimetics, electrocatalytic sensing and energy storage. Mater. Chem. Front..

[38] Sun HQ, Wang YX, Liu SZ, Ge L, Wang L, Zhu ZH, Wang SB (2013). Facile synthesis of nitrogen doped reduced graphene oxide as a superior metal-free catalyst for oxidation. Chem. Commun..

[39] Gogotsi Y, Libera JA, Kalashnikov N, Yoshimura M (2000). Graphite polyhedral crystals. Science.

[40] Ma R, Xia BY, Zhou Y, Li P, Chen Y, Liu Q, Wang J (2016). Ionic liquid-assisted synthesis of dual-doped graphene as efficient electrocatalysts for oxygen reduction. Carbon.

[41] Ma R, Song E, Zhou Y, Zhou Z, Liu G, Liu Q, Liu J, Zhu Y, Wang J (2016). Ultrafine WC nanoparticles anchored on Co-encased, N-doped carbon nanotubes for efficient hydrogen evolution. Energy Storage Mater..

[42] Wang L, Yang C, Dou S, Wang S, Gao X, Ma J, Yu Y (2016). Nitrogen-doped hierarchically porous carbon networks: synthesis and applications in lithium-ion battery, sodium-ion battery and zinc–air battery. Electrochim. Acta.

[43] Wang JC, Senkovska I, Oschatz M, Lohe MR, Borchardt L, Heerwig A, Liu Q, Kaskel S (2013). Imine-linked polymer derived nitrogen-doped microporous carbons with excellent CO_2_ capture properties. ACS Appl. Mater. Interfaces.

[44] Hu F-X, Li L, Lin K, Cui L, Shi C-J, Sayyar AS, Cui S (2016). Preparation of N-doped hollow carbon spheres and investigation of their optical properties. J. Inorg. Mater..

[45] Ma R, Ren X, Xia BY, Zhou Y, Sun C, Liu Q, Liu J, Wang J (2016). Novel synthesis of N-doped graphene as an efficient electrocatalyst towards oxygen reduction. Nano Res..

[46] Wang J, Senkovska I, Oschatz M, Lohe MR, Borchardt L, Heerwig A, Liu Q, Kaskel S (2013). Highly porous nitrogen-doped polyimine-based carbons with adjustable microstructures for CO_2_ capture. J. Mater. Chem. A.

[47] Wei D, Liu YL, Wang Y, Zhang H, Huang LH, Yu G (2009). Synthesis of N-doped graphene by chemical vapor deposition and its electrical properties. Nano Lett..

[48] Xu G-Z, Jin W-W, Zeng X-R, Zou J-Z, Xiong X-B, Huang L, Zhao Z-N (2016). Tailoring of pore structure of coal-based carbon foam. J. Inorg. Mater..

[49] Zhao XH, Gao XP, Zhao HB, Zhang XX, Hao ZX (2016). Highly efficient synthesis of LTA-type aluminophosphate molecular sieve by improved ionothermal method with low dosage of structure-directing agent. J. Inorg. Mater..

[50] Sun LL, Yan K, Luo W, Zhou J (2016). Hollow ZSM-5 zeolite microspheres with improved adsorption and catalytic properties. J. Inorg. Mater..

[51] He W, Jiang C, Wang J, Lu L (2014). High-rate oxygen electroreduction over graphitic-N species exposed on 3D hierarchically porous nitrogen-doped carbons. Angew. Chem. Int. Ed..

[52] Wang JC, Ma RG, Zhou ZZ, Liu GH, Liu Q (2015). Magnesiothermic synthesis of sulfur-doped graphene as an efficient metal-free electrocatalyst for oxygen reduction. Sci. Rep..

[53] Cui H, Yu H, Zheng J, Wang ZJ, Zhu Y, Jia SP, Jia J, Zhu Z (2016). N-doped graphene frameworks with superhigh surface area: excellent electrocatalytic performance for oxygen reduction. Nanoscale.

[54] Lai LF, Potts JR, Zhan D, Wang L, Poh CK (2012). Exploration of the active center structure of nitrogen-doped graphene-based catalysts for oxygen reduction reaction. Energy Environ. Sci..

[55] Zhang Y, Ge J, Wang L, Wang D, Ding F, Tao X, Chen W (2013). Manageable N-doped graphene for high performance oxygen reduction reaction. Sci. Rep..

[56] Aijaz A, Fujiwara N, Xu Q (2014). From metal-organic framework to nitrogen-decorated nanoporous carbons: superior CO_2_ uptake and highly efficient catalytic oxygen reduction. J. Am. Chem. Soc..

[57] Yang HB, Miao J, Hung SF, Chen J, Tao HB (2016). Identification of catalytic sites for oxygen reduction and oxygen evolution in N-doped graphene materials: development of highly efficient metal-free bifunctional electrocatalyst. Sci. Adv..

[58] Iwazaki T, Yang H, Obinata R, Sugimoto W, Takasu Y (2010). Oxygen-reduction activity of silk-derived carbons. J. Power Sources.

[59] Jia Y, Zhang L, Du A, Gao G, Chen J, Yan X, Brown CL, Yao X (2016). Defect graphene as a trifunctional catalyst for electrochemical reactions. Adv. Mater..

[60] Gong FDK, Xia Z, Durstock M, Dai L (2009). Nitrogen-doped carbon nanotube arrays with high electrocatalytic activity for oxygen reduction. Science.

[61] Kim H, Lee K, Woo SI, Jung Y (2011). On the mechanism of enhanced oxygen reduction reaction in nitrogen-doped graphene nanoribbons. Phys. Chem. Chem. Phys..

[62] De Bruijn FA, Dam VAT, Janssen GJM (2008). Review: durability and degradation issues of PEM fuel cell components. Fuel Cells.

